# Maternal antibody and the maintenance of a lyssavirus in populations of seasonally breeding African bats

**DOI:** 10.1371/journal.pone.0198563

**Published:** 2018-06-12

**Authors:** David T. S. Hayman, Angela D. Luis, Olivier Restif, Kate S. Baker, Anthony R. Fooks, Clint Leach, Daniel L. Horton, Richard Suu-Ire, Andrew A. Cunningham, James L. N. Wood, Colleen T. Webb

**Affiliations:** 1 Molecular Epidemiology and Public Health Laboratory (^*m*^EpiLab), Infectious Disease Research Centre, Hopkirk Research Institute, Massey University, Palmerston North, New Zealand; 2 Department of Ecosystem and Conservation Sciences, University of Montana, Missoula, Montana, Montana, United States of America; 3 Disease Dynamics Unit, Department of Veterinary Medicine, University of Cambridge, Cambridge, United Kingdom; 4 Institute for Integrative Biology, University of Liverpool, Liverpool, United Kingdom; 5 Wildlife Zoonoses and Vector-borne Diseases Research Group, Animal and Plant Health Agency (APHA), New Haw, Surrey, United Kingdom; 6 Department of Clinical Infection, Microbiology and Immunology, Institute of Infection and Global Health, University of Liverpool, Liverpool, United Kingdom; 7 Department of Biology, Colorado State University, Fort Collins, Colorado, United States of America; 8 School of Veterinary Medicine, University of Surrey, Guildford, Surrey, United Kingdom; 9 Wildlife Division of the Forestry Commission, Accra, Ghana; 10 Institute of Zoology, Zoological Society of London, Regent’s Park, London, United Kingdom; Wistar Institute, UNITED STATES

## Abstract

Pathogens causing acute disease and death or lasting immunity require specific spatial or temporal processes to persist in populations. Host traits, such as maternally-derived antibody (MDA) and seasonal birthing affect infection maintenance within populations. Our study objective is to understand how viral and host traits lead to population level infection persistence when the infection can be fatal. We collected data on African fruit bats and a rabies-related virus, Lagos bat virus (LBV), including through captive studies. We incorporate these data into a mechanistic model of LBV transmission to determine how host traits, including MDA and seasonal birthing, and viral traits, such as incubation periods, interact to allow fatal viruses to persist within bat populations. Captive bat studies supported MDA presence estimated from field data. Captive bat infection-derived antibody decayed more slowly than MDA, and while faster than estimates from the field, supports field data that suggest antibody persistence may be lifelong. Unobserved parameters were estimated by particle filtering and suggest only a small proportion of bats die of disease. Pathogen persistence in the population is sensitive to this proportion, along with MDA duration and incubation period. Our analyses suggest MDA produced bats and prolonged virus incubation periods allow viral maintenance in adverse conditions, such as a lethal pathogen or strongly seasonal resource availability for the pathogen in the form of seasonally pulsed birthing.

## Introduction

Understanding what mechanisms allow maintenance of infections within populations is fundamental to disease ecology [1, 2]. Pathogens that cause acute disease and death or lasting immunity may require specific spatial (e.g. metapopulation [3]), or temporal (e.g. hibernation [4]) processes or multiple host species to persist in populations [5]. Lyssaviruses, such as rabies virus (RABV), cause acute disease that is invariably fatal in most mammalian species once clinical signs develop. Models of lyssavirus dynamics have concentrated on RABV in terrestrial mammals (e.g., domestic dogs, raccoons and foxes [6–11]). However, the vast majority of lyssaviruses, of which RABV is only one, have bats as their natural (co-evolved) reservoir hosts [12–14]. Phylogenetic analyses suggest that all lyssaviruses, including RABV, originated from bats [15]. The genetic distance among some African and Eurasian bat lyssaviruses means that RABV-derived vaccines are ineffective in providing protection due to a lack of cross-reactivity [16–18]. Understanding the dynamics of bat lyssaviruses is therefore necessary to understand infection emergence and to manage zoonotic disease risk [19–22].

Bats are long-lived mammals with typically highly synchronous birthing [23, 24]. A study of RABV in big brown bats (*Eptesicus fuscus*) in Colorado used a mathematical model with data from field studies to provide support for the hypothesis that life history patterns, such as seasonally variable mortality rates, allow for RABV maintenance in temperate zone bats [4]. Overwinter hibernation by bats typically reduces bat mortality. Bat hibernation causes RABV replication to slow or cease, thus extending the incubation period throughout the duration of overwintering [4, 12]. RABV has long viral incubation rates anyway, and, together, the overwinter extension and long incubation periods were necessary in the RABV- *E*. *fuscus* model for RABV persistence at the population level [4]. This is because these factors allow virus to persist in the population until naïve young are born in the spring. The importance of reduced overwinter mortality during hibernation to RABV maintenance in this temperate system prompts the question; what mechanisms allow for viral maintenance in species in tropical regions that do not hibernate?

Anti-lyssavirus antibodies are detected in many bat species. In a study of RABV in tropical vampire bats (*Desmodus rotundus*), models fit to spatially replicated, longitudinal field seroprevalence data supported findings that most RABV exposures are non-fatal and concluded dispersal was necessary to allow RABV persistence at the population level [25]. These studies suggest spatially or temporally variable resources that determine host population dynamics may be driving RABV dynamics. However, in the vampire bat system, birthing occurred throughout the year but with seasonal oscillations (modelled with a cosine function). Another *Lyssavirus*, Lagos Bat Virus (LBV), is endemic in African fruit bats. LBV has not been reported as a cause of human death, but it has fatally infected a range of non-bat species [26, 27] and little is known about the factors enabling its maintenance [28–30]. In contrast to temperate bats, the lack of hibernation in African fruit bats, combined with sharp, highly synchronous birth pulses, would be expected to favour the rapid extinction of the virus in small populations. Yet, Peel *et al*. found seropositive bats on Annobon in a small (<2,500) isolated population [31–33], and seropositive bats are found across the continental range of *E*. *helvum* [29, 32–37].

In addition to seasonal birthing [2, 38, 39], there has been increased interest in how other host-related factors, including maternally-derived antibody (MDA) [40–42], affect pathogen infection dynamics and maintenance within populations [43]. Investigations into the role of the transient protection offered by MDA suggest that immune young delay transmission, potentially affecting infection dynamics in natural populations [40]. Long-lived species are expected to invest more in antibody production and are more likely to have functional MDA [41]. A recent model including strong seasonal birthing suggests demographic turnover, infection duration, and pre-existing immunity interact with birth pulses to determine pathogen maintenance beyond the initial epidemic [39]. New analyses of field serological data strongly suggest MDA and lifelong immunity against LBV infection in wild *E*. *helvum* populations [44].

To better understand these field serological data we measure LBV antibody decay in a captive colony of *E*. *helvum*. We hypothesise that duration of immunity, combined with slow infection dynamics, may be key factors in the maintenance of LBV within closed populations of bats. To test this hypothesis, we incorporate epidemiological, immunological and ecological data on LBV in *E*. *helvum* into a stochastic disease dynamic model. We use a range of sensitivity analyses to help elucidate the host and pathogen traits that allow LBV infection to persist in *E*. *helvum* populations.

## Methods

### Ethics statement

Ethical approval for this project (WLE/0467) was received from the Zoological Society of London Ethics Committee and locally from the Ghanaian Veterinary Services Directorate and Wildlife Division of the Forestry Commission, Ghana.

### Location

All field data were collected, and sampling was undertaken, in Ghana, West Africa. These data comprised serological data from 1167 bats, including 322 juvenile and 845 adult bats, collected during 12 sampling sessions over a four-year period [32, 34, 36].

### Captive study

We performed a study on captive bats in Ghana [45]. Bats classified by age (neonate, juvenile, sexually immature adult, and sexually mature adult) and sex (male and female) were bled on entry and at recapture approximately every 2–4 months. Criteria for assessing age and reproductive status are fully described in Peel *et al*. [32]. Serological data available for 91 individual captive bats are reported in this study and Method A in S1 Supplementary Information.

### Laboratory studies

The presence of LBV infection from oropharyngeal swab samples from all tested bats was determined using a pan-lyssavirus SYBR^®^ Green (Applied Biosystems) real-time reverse transcription PCR (RT-qPCR) able to detect as low as 25 LBV copies [46]. RNA extraction from frozen tissues was by MELT^™^ (Ambion) and KingFisher 96^®^ (Thermo Electron Corporation). RNA was reverse-transcribed and analysed using RT-qPCR. All samples were tested by 18S RT-qPCR to ensure RNA was extracted successfully and LBV positive and negative controls were used with each extraction and PCR.

Anti-LBV virus neutralising antibody (VNA) titer was used as a proxy for bat immunity [47]. Serological testing for VNA was by modified fluorescent antibody virus neutralisation test (mFAVN). Briefly, the LBVNig56 isolate [48] was used, because it was isolated from the region (Nigeria) from an *E*. *helvum* bat and LBV has been shown to be genetically similar in locations across time [49] (see Discussion). Negative (dog) and two positive (rabbit) control sera from Horton *et al*., [18] were included as known negative samples in the absence of an available known negative *E*. *helvum* serum sample [50].

Reciprocal serology titers were calculated using the Spearman-Karber equation. This requires the number corresponding to the last dilution of sera that neutralises all four of its replicates (wells), i.e. four negative results in a column, to be used to find the end-point titre. We used a 2-fold, rather than 4-fold, dilution to attempt to obtain higher resolution antibody decay data. Captive bats were considered seropositive if the serum sample neutralised LBV at a reciprocal titer of greater than 11.3, the lowest titer that could be tested due to the 2-fold dilution series and the Spearman-Karber equation. For comparison, the RABV FAVN 0.5 IU/ml standard required to ensure an adequate vaccination response routinely neutralises around a reciprocal titer of 16 to 20, thus our threshold is deliberately sensitive by comparison but clearly distinguishable from negative and non-specific neutralisation. All samples from individual bats were tested on the same plate to ensure that inter-test variation did not affect antibody titer measurement. Cross-sectional, longitudinal seroprevalence data are reported elsewhere [29].

### Data analysis

#### Serological data

To estimate antibody titer decay rates from captive bats, the effect of time on antibody titers was estimated using binary log_2_-transformed antibody titer data and linear mixed-effects (lmer) regression models [51, 52]. We addressed individual effects of different initial serological titers by using individuals as a random categorical variable for each age category, thus the model was:

$${Log}_{2}Titer=\beta_{0}+\beta_{day}X_{day}+\beta_{age}X_{age}+\beta_{day:age}X_{day:age}+\mu +\epsilon$$

Where *day* is day of captivity, *age* age-class, *μ* random individual intercepts, and ε the error. We used the sjPlot and ggplot2 R packages for plotting results [53, 54].

### Population dynamic model

**Fig 1:**
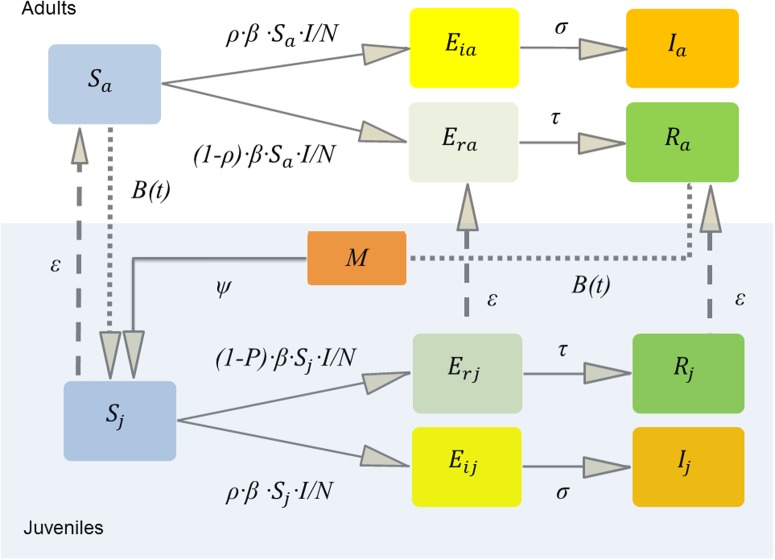
Schematic of the model structure. The total bat population is *N;* susceptible *S;* exposed *E;* infectious *I;* immune *R;* the proportion becoming infectious *ρ*; exposed leading to infection *E*_*i;*_ exposed leading to recovery *E*_*r;*_ adults _*a*_ (top)_*;*_ juveniles _*j*_ (bottom, shaded). Juveniles age at rate *ε* (see text for *M* aging); maternally-derived antibody positive juveniles *M;* having waning immunity at rate *ψ*; and juveniles are born with a seasonal birth pulse *B(t)*. Lagos bat virus transmission was frequency dependent *β·S·I/N*. *E*_*i*_ become infectious at rate σ (1/incubation period). *E*_*r*_ seroconvert at rate τ (1/seroconversion period). Mortality is omitted for clarity.

To model the population dynamics with different adult and juvenile mortality rates and to incorporate MDA and seasonal birthing [2, 39] we constructed an age-structured model. Field and laboratory studies confirm that in some populations only a proportion of bats become infectious following lyssavirus exposure, while other mount an immune response detectable through serological assays [4, 14, 25, 55]. We therefore developed a compartmental model of the bat population (*N*), comprised of susceptible (*S*), exposed (*E*), infectious (*I*) and immune (*R*) classes, for the *E*. *helvum*-LBV system (Fig 1). We assumed LBV transmission was frequency dependent, thus, susceptible (*S*) individuals became exposed at the rate (*β·S·I/N*) [56]. A proportion (*ρ*) of the bats that had the virus transmitted to them incubated the virus to become infected and infectious and died. Those exposed and incubating the virus (*E*_*i*_) became infectious at rate *σ* (1/incubation period). After the incubation period, the bats became infected and infectious (*I*) before dying of disease induced mortality at rate *α* (1/infectious period). The remaining proportion (1-*ρ*) mounted an immune response (*E*_*r*_) at rate τ (1/seroconversion period) and survived as immune adults (*R*). We assumed immune adults were immune for life, based on field data [44] and supported by our captive study results (see below). We included density-dependent mortality with different juvenile (*δ·N/K*) and adult (*μ·N/K*) rates [2, 23]. We assumed density dependent mortality occurs in all classes except those that die of disease induced mortality at rate *α*, because the disease causes death within days. Births (*b*, see below) were not density dependent, as pregnancy rates estimated from field studies approach 100% [23, 57]. Susceptible (*S*_*a*_), exposed and seroconverting (*E*_*ra*_), and immune (*R*_*a*_) adults bred annually with a seasonal birth pulse, but not those incubating infection (*E*_*ia*_) or those that were infectious (*I*_*a*_). We made this distinction because becoming infectious with lyssavirus is invariably fatal in bats, and maturation of dependent neonates was assumed to be slower than the incubation and infectious period of disease in the mother. Those born to immune adults (*R*_*a*_, at the same rate as those born to *S*_*a*_) had maternally-derived antibody (*M*), which waned at rate *ψ* and controlled the transition to a susceptible juvenile class. To ensure *M* class bats did not extend the mean time juveniles spent in the *S*_*j*_ class, bats from the *M* class aged into a separate *S*_*jm*_ class, before aging to *S*_*a*_ at rate *γ*, whereas those born directly into the susceptible class, *S*_*j*_ (from *S*_*a*_) aged at rate *ε* (1/365). Thus, the mean time in days, *d*, spent in the *M* class led to the rate *ψ* = 1/*d* and time spent in the *S*_*jm*_ class 365-*d*, thus *γ* = 1/(365-*d*). Juveniles that became infectious (*E*_*ij*_ and *I*_*j*_) also did not mature in our model, because the clinical disease (rabies) is expected to be fatal and juveniles will not be able to age to adults and breed. For a schematic of this model see Fig 1. The ODE model structure is:

$$\frac{{dS}_{j}}{dt}=-\frac{\beta S_{j}(I_{j}+I_{a})}{N}+b(S_{a}+E_{ra})-\delta S_{j}\frac{N}{K}-\varepsilon S_{j}$$

$$\frac{{dM}_{j}}{dt}=bR_{a}-\delta M_{j}\frac{N}{K}-\psi M_{j}$$

$$\frac{{dS}_{jm}}{dt}=-\frac{\beta S_{jm}(I_{j}+I_{a})}{N}-\delta S_{jm}\frac{N}{K}-\gamma S_{jm}+\psi M_{j}$$

$$\frac{{dS}_{a}}{dt}=-\frac{\beta S_{a}(I_{j}+I_{a})}{N}+\varepsilon S_{j}+\gamma S_{jm}-\mu S_{a}\frac{N}{K}$$

$$\frac{{dE}_{ij}}{dt}=\rho \frac{\beta S_{j}(I_{j}+I_{a})}{N}+\rho \frac{\beta S_{jm}(I_{j}+I_{a})}{N}-\delta E_{ij}\frac{N}{K}-\sigma E_{ij}$$

$$\frac{{dE}_{ia}}{dt}=\rho \frac{\beta S_{a}(I_{j}+I_{a})}{N}-\mu E_{ia}\frac{N}{K}-\sigma E_{ia}$$

$$\frac{{dE}_{rj}}{dt}=(1-\rho )\frac{\beta S_{j}(I_{j}+I_{a})}{N}+(1-\rho )\frac{\beta S_{jm}(I_{j}+I_{a})}{N}-\varepsilon E_{rj}-\delta E_{rj}\frac{N}{K}-\tau E_{rj}$$

$$\frac{{dE}_{ra}}{dt}=\frac{\left(1-\rho )\beta S_{a}(I_{j}+I_{a}\right)}{N}+\varepsilon E_{rj}-\mu E_{ra}\frac{N}{K}-\tau E_{ra}$$

$$\frac{{dI}_{j}}{dt}=\sigma E_{ij}-{\alpha I}_{j}$$

$$\frac{{dI}_{a}}{dt}=\sigma E_{ia}-\alpha I_{a}$$

$$\frac{{dR}_{j}}{dt}=\tau E_{rj}-{\delta R}_{j}\frac{N}{K}-\varepsilon R_{j}$$

$$\frac{{dR}_{a}}{dt}=\tau E_{ra}-\mu R_{a}\frac{N}{K}+\varepsilon R_{j}$$

### Stochastic model

In order to explore the role of demographic stochasticity in the persistence of the virus in the population, we implemented the above model as a continuous Markov process in the “pomp” R package [58]. We coded the model in native C code and modeled births as a Poisson process and the remaining processes with Euler multinomial processes [2], such that *N* individuals face a constant hazard to leave the class in *k* ways, at rates *r*_*1*_, *r*_*2*_, *…r*_*n*_, then during *Δt* time interval, the number of individuals is multinomially distributed:

$$(N-\sum_{i=1}^{k}\Delta n_{i},\Delta n_{1},\ldots ,\Delta n_{k})\sim multinomial({N;p}_{0}p_{1},\ldots {,p}_{k}),$$

Where *Δn*_*i*_ is the number of individuals leaving in way *i* over the time interval and the probability of remaining is *p*_0_ = exp(−∑_*I*_ *r*_*i*_Δt) and of leaving in way *j* is $p_{j}=\frac{r_{j}}{\sum_{i}r_{i}}(1-exp\;(-\sum_{i}r_{i}\Delta t))$.

### Parameter estimation

#### Birth rates

The birth rate for *E*. *helvum* is seasonally pulsed [23, 57]. We previously described the use of a periodic Gaussian function (PGF) that allows births to be pulsed over a period and ensures that no births occur outside the birthing season, *B*(*t*) = *κexp*[−*scos*^2^ (*π*t − *φ*)] [2, 39]. Time in days is *t*, *s* controls the width of birth peaks (“synchrony”), φ the phase of the pulse, and *κ* is a scaling parameter that controls the height of the peak. We then fit the PGF using the Nelder-Mead optimization function *optim* in R [52] and fixed the shape parameters to estimate κ, such that the integral of the PGF (the annual birth rate) equalled the birth rate estimated from field data [23] (Figure A in S1 Supplementary Information).

#### Death rates

We previously estimated annual survival probability from capture-recapture data for *E*. *helvum* from a wild colony in Accra, Ghana; this showed that an exponential function with constant survival across adult ages was the best model [23, 59]. We used the estimated annual survival rate following the transformation of survival rates [2], thus $Annual\;rate=-\frac{\left[ln(1-S)\right]}{t}$ where *S* is the apparent annual survival probability and *t* time.

### Infection parameters

All host parameters were from *E*. *helvum*, however, the LBV-infection related parameters, seroconversation rate, natural incubation period and infectious period, were not available for LBV in bats. For those, we assumed consistency across bat lyssaviruses; we took parameter values from other bat-RABV studies. We then used our dynamic model to estimate two LBV parameters, *β* and *ρ*, that may be inconsistent with RABV (Table 1). We also tested three alternative assumptions about force of infection (FOI) across age classes, by fitting three models to age-specific serological data: an age independent model, a model of increasing or decreasing FOI with age and a higher order polynomial function that allows flexible curves and thus variable FOI.

**Table 1:** Model variables and parameters for the Lagos bat virus and *Eidolon helvum* system.

Variable/Parameter	Symbol	Value	Unit	Reference	Range used in sensitivity analysis
**Transmission rate (frequency dependent)**	β	7.635	Per capita per day^2^	This study	0.76–76
**Juvenile mortality rate**	δ	2.31×10^−3^	Per capita per day	[23]	2.31×10^−4^ to 2.31×10^−2^
**Adult mortality rate**	μ	5.1×10^−4^	Per capita per day	[23]	5.1×10^−5^ to 5.1×10^−3^
**Disease induced mortality rate**	α	0.2	Per capita per day	[83]	2×10^−2^ to 0.99
**Probability of becoming infectious once transmission occurs**	ρ	0.056	-	This study	1×10^−2^ to 0.99
**Rate of aging from juvenile to sexually mature adult**	ε	2.7×10^−3^	Per capita per day	[23]	NA, as field data suggest bats breed in second year of life [23]
**Rate of aging from juvenile to sexually mature adult for formerly maternally-derived immune juveniles**	γ	3.8×10^−3^	Per capita per day	[23]	NA, as field data suggest bats breed in second year of life [23]
**Incubation rate (or 1/ incubation period)**	σ	2.1×10^−2^	Per capita per day	[4]	2.1×10^−3^ to 0.2
**Rate of seroconversion**	τ	4.2 ×10^−2^	Per capita per day	[4]	4.2×10^−3^ to 0.42
**Rate of loss of maternally-derived immunity**	ψ	0.01	Per capita per day	This study	2.74×10^−3^ to 0.1
**Parameter to control timing of births**	φ	0	-	[2]	NA
**Annual birth pulses**	ω	1/365	Per year	[23]	NA as field data show only one young per female per year [23]
**Scalar to control birth rate**	κ	1.5/365	Per female per day	[2]	NA
**Annual birth synchrony**	s	14.35	-	[2]	1.435 to 143.5
**Carrying capacity**	K	1×10^6^	-	[23]	1×10^4^ to 1×10^6^

### Model fitting and estimation of unknown infection-related parameters

We fitted the model to mean estimates of juvenile and adult wild bat seroprevalence available from the longitudinal field study in Ghana [29] and estimated the likelihood of the data given the model and the parameter set θ (i.e. *β* and *ρ*) using the sequential Monte Carlo particle filter function in the partially observed Markov processes (‘pomp’) package in R [58]. The number of seropositive individuals *k* was given by *k* ∼ binomial(*n*, *p*), where *n* was the number of samples collected, and *p* was the seroprevalence predicted by the process model for a given parameter set *θ*. Therefore, for seropositive adults (_*a*_), for sampling times *t = 1*, *2 … 12*, the model was *k*_*a*,*t*_ ~ *binomial*(*n*_*a*,*t*_, *p*_*a*,*t*_). A similar binomial model for juveniles was used. We used 1000 particles and summed the conditional log likelihoods for our final likelihood estimate. We fit the model to the data after allowing the model to run for ~ 20 years to allow the system to equilibrate to regular oscillatory dynamics. Alternative FOI models were compared using Akaike information criterion (AIC, see Method B in S1 Supplementary Information).

### Model validation

We calculated the correlation between the predicted mean adult and juvenile seroprevalence results from 1000 model simulations using the MLE for the infection parameters and the point estimates from the data (in-sample testing). Because a low sample size precluded good out-of-sample prediction, we used jackknifing, where we removed a single time point data pair (i.e. both adult and juvenile seroprevalence) and re-estimated the infection-related parameters and the final seroprevalence estimates to determine the sensitivity of our outcomes to individual data points.

### Pathogen extinction—Sensitivity analysis

Given the uncertainty of some parameters and to examine the relative importance of biological aspects of the system, such as MDA, on infection maintenance, we performed a sensitivity analysis [60]. We constructed 100 stratified, random parameter sets for the parameters of interest using Latin hypercube sampling [61, 62]. Parameters were varied +/- an order of magnitude, with the exception of those for which this would be outside biologically plausible values (Table 1). Each of the 100 parameter sets was simulated 1000 times for 35 years. Running the model for 35 years allowed the system to equilibrate to regular oscillatory dynamics for all parameter combinations and ensured that varying *K* did not cause density dependent mortality to approach zero at small population sizes. Partial-rank correlation coefficients (PRCC) between each parameter and the persistence of infection in the population after 35 year simulations determined the relative importance of each parameter [2, 62].

## Results

### Captive bat study

#### Molecular studies

Lyssavirus RNA was not detected by RT-qPCR from the throat swabs of any bat on entry to the captive colony, nor was lyssavirus detected in the brains of nine captive bats that died during the study period. All LBV positive controls were positive and host 18S RNA was detected in all samples (but not negative controls), confirming successful RNA extraction and RT-qPCR. We therefore assumed that no LBV virus circulation occurred within the colony on the assumption that productive infection always leads to death.

#### Serological studies

**Fig 2:**
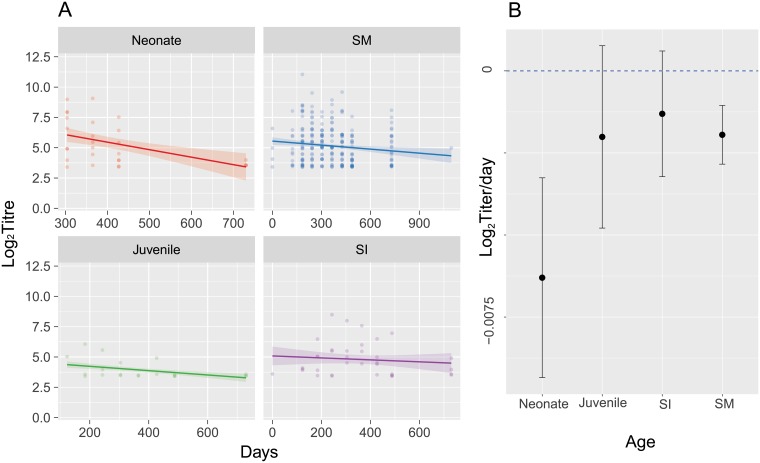
Captive bat anti-Lagos bat virus antibody titers and daily changes in titer. The regression analysis of longitudinal captive bat anti-LBV antibody titers was adjusted for inclusion of multiple data points from the same individual. The ages are the ages at which the bats entered the study. All neonates were born in captivity. Time series with mixed effects model predictions (A) and mean decay rate (B, regression coefficients) with 95% confidence intervals are plotted. Note the different x-axes scales. Raw data are in S1 Dataset.

Anti-LBV VNA titers were available for 60 sexually mature adults, 10 sexually immature adults, 8 juveniles, and 13 neonate captive individuals, with 10/13 neonates born seropositive. Of 8 known mother-offspring pairs in which the offspring were seropositive, 7 had seropositive mothers. One single mother did not test seropositive during the study, although the serological status and infection history of the animal before captivity is unknown. Two of the 7 seropositive mothers did not remain seropositive throughout. One of those tested positive on entry with a low reciprocal titer (16), lost detectable antibody and did not re-seroconvert, and the other was seronegative on entry, but fluctuated to a low positive (reciprocal titer 16) and back to negative twice during the course of the study. One neonate of unrecorded parentage born seronegative then had a detectable titer on two subsequent tests 2 and 4 months after the first sample, but then became seronegative. All seropositive neonates were estimated to have their antibody titer decayed to our negative threshold by 1.28 years, but with very wide 95% confidence intervals (0.46–4.18 years 95% CI). Adult bat antibody titers were estimated to decay to our negative threshold by 3.8 years (2.9–6.7 95% CI) (Fig 2). As the estimated mean ages for this species ranges from 2.3 to 6.7 years [59], for the purposes of our model, we assume bats to be immune for life [44]. Levels of antibody fluctuate naturally over time [41], and therefore for those near the seropositive threshold (reciprocal titer 11.3), seropositivity may fluctuate (Figure B in S1 Supplementary Information). More generally, the distribution of presumably infection related titers from captive juvenile, sexually immature and mature adult bats was similar to those of wild bats from the same population (Figures C and D in S1 Supplementary Information). Thus, for our model we assume that the presence of neutralising antibody at any time point meant immunity. Raw data are in S1 Dataset.

### Parameter estimation

The best fitting parameters for birth pulse synchrony (*s*) and the corrected *κ*, the scalar for the PGF birth pulse, are given in Table 1 and Figure A in S1 Supplementary Information. The integral of the function was equal to the annual birth rate (0.49) that we reported for *E*. *helvum* [23].

Our analysis of the age-specific serological data suggests FOI is age independent (AIC weight (*w*AIC) = 0.58), or at least that this simplifying assumption was reasonable (Figure E in S1 Supplementary Information, other model *w*AIC were 0.3 and 0.12).

The MLE for the parameters *β* (transmission coefficient) and *ρ* (the probability of becoming infectious) were 0.056 and 7.635 respectively (Table 1, Figure F in S1 Supplementary Information). The infection dynamics using these parameters and the stochastic model are shown in Figures G–I in S1 Supplementary Information.

### Model validation

The correlation between the mean seroprevalences from 1000 model simulations and the data (in-sample testing) was reasonable (R = 0.74, 0.48–0.88 95% CI, t = 5.1, df = 22, p-value = 3.8×10^−5^; Figure I in S1 Supplementary Information). When we used jackknifing to perform cross-validation, the correlation between model results and the data was noisy, but reasonable (R = 0.60, 0.25–0.81 95% CI, p-value = 0.0021, Figure J in S1 Supplementary Information). The parameter estimates of *β* and *ρ* were sensitive to removal of these data points and between two parameter spaces (Figure K in S1 Supplementary Information). Due to the correlation between these parameters, however, the actual model simulation results were insensitive to these changes.

### Sensitivity analysis

**Fig 3:**
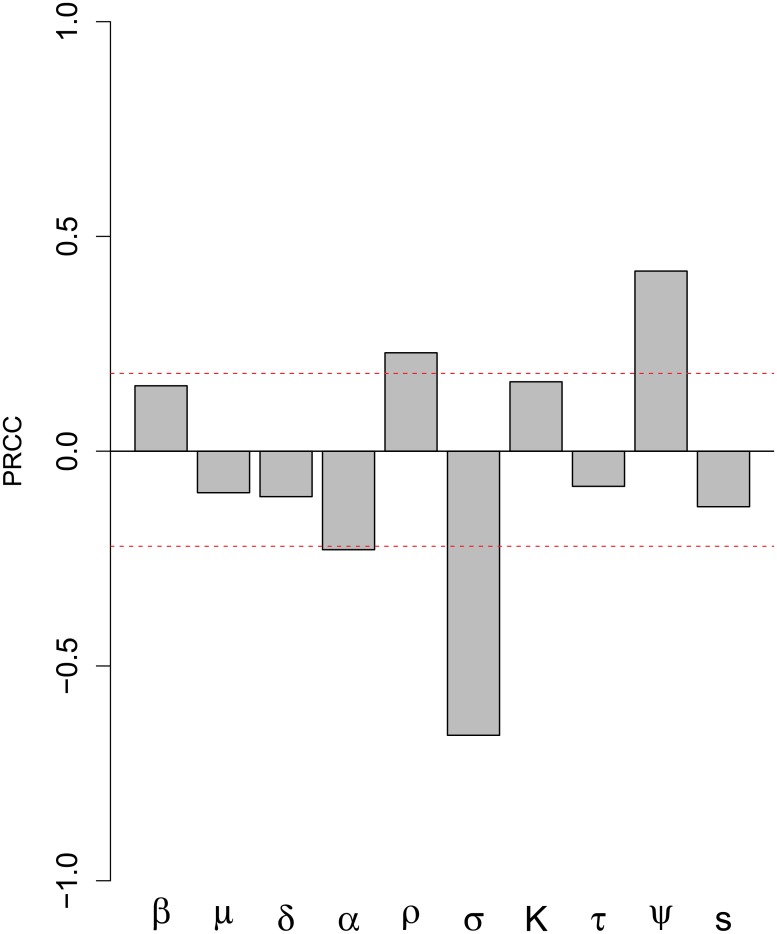
Sensitivity analysis results. Partial rank correlation coefficients (PRCC) for infection maintenance for 1000 simulations for 100 parameter sets for a 25 year time period for our stochastic *Eidolon helvum*–Lagos bat virus model. Positive PRCC indicate increasing a parameter increases infection maintenance. Parameters are: transmission rate *β*; adult mortality rate *μ*; juvenile mortality rate *δ*; disease induced mortality *α*; probability of becoming infectious *ρ*; incubation period *σ*; carrying capacity *K;* rate of seroconversion *τ*; rate of loss of maternally-derived immunity *ψ*; and annual birth synchrony *s*. Significance at α = 0.05 is demarcated by the red line. Parameters were varied according to those ranges in Table 1.

PRCC sensitivity analysis results suggest that LBV maintenance is significantly positively affected by increasing infectivity (parameter *ρ*, the proportion exposed becoming infectious) and MDA decay rates (*ψ*) (Fig 3). LBV maintenance is decreased by reducing the incubation period (i.e. increasing the transition rate, *σ*, from exposed to infectious) and, to a lesser extent by the disease-induced death rate (α). The latter parameter determines the average duration of the infectious period (1/α) because in our model all infectious bats die of clinical disease.

**Fig 4:**
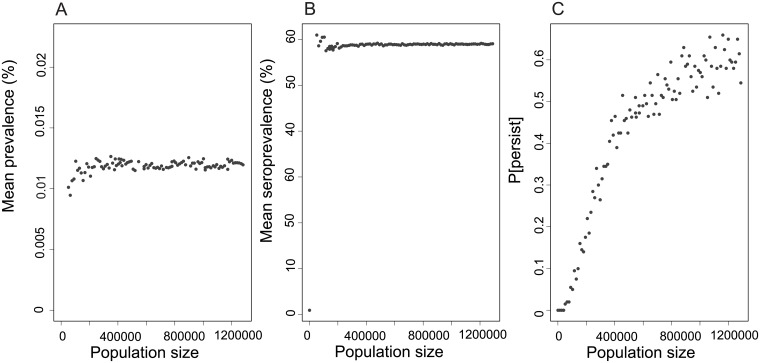
Predicted Lagos bat virus metrics. (A) Prevalence (%), (B) seroprevalence (%), and (C) maintenance (proportion) in an *Eidolon helvum* population model with maternally-derived antibody, age structure (juvenile and adult), and a seasonal birth pulse. Parameters are as Table 1, but with the carrying capacity parameter, *K* to control the population size for each age class. The mean prevalence (A) and seroprevalence (B) were estimated from the results of each of 1000 simulations at the end of 25 years of simulation, excluding the runs in which the infection did not persist (C).

Our sensitivity analysis confirmed that the probability of population level persistence was positively associated with the carrying capacity *K* (a proxy for colony size). For example, in simulations of our best-fit model, with a colony size around 600,000, LBV persisted in approximately 50% of 35-year simulations; the probability of maintenance plateaus at approximately 60% of simulations when populations reach a size of approximately 1,000,000 (Fig 4).

When infection is maintained in populations, the prevalence of infectious bats remains low (approximately 0.012%, range 0.009–0.013%) despite increasing population sizes. The same responses are seen in the seroprevalence results, which plateau at around 60% in populations within which the infection persists. The overall seroprevalence including simulations in which the infection fails to persist is 30–40%, but increases with the probability of maintenance. Given the infection prevalence plateaus at approximately 0.012%, this implies that very few infectious individuals are present in any population at any point in time, despite high seroprevalence, with this value suggesting approximately 1 in 8,300 animals being infected at any time in large populations. Thus, it is not surprising that we did not find any individuals with an active infection in our study of 1167 wild bats, of which 796 were tested for viral RNA [29].

## Discussion

Our study enabled us to confirm that MDA to LBV in *E*. *helvum* exists and to estimate antibody rates of decay in captive bat (Fig 2). Our results support field data that suggest adult infection-derived antibody duration is prolonged and perhaps lifelong [44]. Combining species-specific field serological and demographic data into a mechanistic model of LBV dynamics, we predict that MDA may reduce the probability of virus maintenance in populations of these tropical bats (Fig 4).

Our captive bat study suggest that there is substantial variation in antibody titers through time, with antibody decay rates being most rapid in neonates. As no LBV infection was detected during the study period in the captive colony we assume that no re-exposure would have occurred to boost antibody responses during the study. We therefore assume the antibody dynamics detected in juvenile, sexually immature and mature adults resulted from pre-capture exposure.

Our estimate that bats had lost MDA by 1.28 years, but with wide 95% confidence intervals (0.46–4.18 years), broadly supported field studies of LBV in *E*. *helvum* that show a dip in seroprevalence at 11–12 months of age [44]. Studies have found MDA are typically lost by 12 months in bats, but generally within the first 6 months [63–65]. Our MDA duration estimates, however, are considerably longer than mean field estimates (3.7–8.1 months). This could be due to a number of factors. Studies have reported Pteropodidae bats have high IgG levels in milk, with data suggesting the majority of IgG is transferred through milk rather than the placenta for another Pteropid bat species [66]. In our captive colony, birthing may be less synchronous than in the wild, and this along with the possibility of communal nursing could facilitate prolonged acquisition, and hence persistence, of MDA in captivity [67–69]. Communal nursing might also explain how a seropositive neonate was found with a seronegative mother. Future studies with increased sampling frequency and sample size are needed to confirm our results and to determine the biological function of lyssavirus MDA.

Our results support field data that suggest infection-induced antibody titers decay more slowly than MDA [44]. We found that low titers fluctuate around the negative cut-off level used to determine if animals are seropositive or not, similar to challenge studies in bats [55]. The slow antibody decay rate is supported by findings in humans, in which antibodies against some RNA viruses can be long-lived [70]. Our estimates of the duration of antibody persistence (3.8 years, 2.9–6.7 95% CI) in the captive bats are lower than field estimates (12 years, 5.2 –infinite 95% CI) [44]. This might be due to wild bats being re-exposed to LBV as the virus circulates, compared to the captive bat colony in which there was no molecular or clinical evidence of circulating LBV.

Given that we measured virus neutralising antibody and we have estimated mean *E*. *helvum* life expectancy to be 2.3 to 6.7 years [23, 59], we assumed lifelong immunity from infection-derived (but not maternally-derived) antibody. Whether these virus neutralising antibodies are protective or not against LBV challenge is unknown [71]; in challenge studies bats with detectable anti-RABV titers have succumbed to experimental RABV infection [55]. Despite generally declining antibody titers, substantial within-bat variation in titers through time was found even in samples from individuals being tested on one 96 well plate with the same virus titers to reduce variation. This variation is within the usual variation expected for the virus neutralisation test, as noted previously [18, 50], and might reflect dynamic processes related to the bats (dynamic immune responses) and the test (dynamic cell growth and viral replication). An absence of LBV isolation or detection throughout the period, and the more rapid loss of antibody in the captive bats compared to in the wild, leads us to believe these dynamic antibody responses are not due to infection. We acknowledge, however, we cannot conclusively state that no virus was present or that intermittent oral shedding of virus and recovery from infection did not occur in our captive colony.

Recent studies have reported an LBV isolate from Ghanaian *E*. *helvum* [30]. Interestingly, this virus was LBV lineage A and was found to induce lineage-specific neutralizing antibodies [30] and more rapid death in intracerebrally infected bats [72]. In our study, we used the LBVNig56 isolate [48] because it was available from the beginning of our study, was isolated from the region (Nigeria), was isolated from an *E*. *helvum* bat, and genomic studies in South Africa have shown LBV isolates obtained 25 years apart have 98.9–99.9% nucleotide and 99.1–100% amino acid identity [49]. The conserved genome of LBV through time [49] and the lack of cross-reactivity found by Freuling *et al*. [30], indicate that we observed the dynamics of antibody only to a lineage B virus, even if a lineage A virus is circulating in our study population. Further studies are required to determine the degree of cross-reactivity among these viruses, along with efforts to isolate more viruses.

The estimated probability of a bat becoming infected, infectious, and thus dying from LBV infection (*ρ*) is low, 3–6% (Figure F in S1 Supplementary Information). This estimate is lower than values for RABV in *E*. *fuscus* in the US (15%, [4]) and vampire bats (*D*. *rotundus*) in Peru (10%, [19]). Whether this reflects a true difference is unknown as in each case the data available to estimate this parameter are sparse, and there is another area of parameter space with higher *ρ* (15% infected) and lower *β* that also has reasonable support and produces similar infection dynamics. This reflects an identifiability issue between *ρ* and *β*, as jackknifing moved the best estimates to between these two parameter spaces. Evolutionary models suggest RABV has a more recent ancestry than LBV [13, 15], thus *E*. *helvum* may have had longer to adapt to LBV than American bats to RABV, which could explain the lower *ρ* in our system. Notably, increasing the proportion of bats that become diseased and transmit virus (*ρ*) increases viral maintenance in the sensitivity analysis.

Our field data parameterised model suggests that infection prevalence and seroprevalence in populations plateau at around 0.012% and 60% respectively (excluding simulations where infection did not persist). These results could explain two seemingly contradictory patterns. Firstly, across Africa, LBV infection appears widespread. The seroprevalence against LBV in *E*. *helvum* across ten sub-Saharan African countries ranged from 5.8 to 80 percent [32, 33], but the first, second (median) and third quartiles are 32, 37 and 40% respectively (Figure L in S1 Supplementary Information), suggesting that seroprevalence is remarkably similar across the species’ distribution, as well as through time [29]. This is in concordance with our model predictions. Secondly, infection has been difficult to detect [28–30] and spillover events are rare [26, 27, 49], which is also consistent with the very low prevalence predicted by our model. The low infection prevalence and use of a model with frequency dependent infection are supported by other lyssavirus studies. For example, low infection prevalence was estimated for vampire bat rabies [25], while studies of domestic dog RABV suggest the basic reproductive number (*R*_*0*_) for rabies is low (<2), irrespective of the dog population density [73].

Our modelling study might help explain the low seroprevalence in a small *E*. *helvum* population on the island of Annobon [31]. Our model predicts LBV may persist in small populations with no clear threshold below which infection fails to persist, even though maintenance is unlikely in small populations [74]. The model results suggest that in all populations LBV prevalence remains low and relatively constant. However, for those populations in which the virus does persist, seroprevalence remains high, in contrast to the reports from Annobon. This contradictory result might be accounted for if LBV fails to persist in smaller meta-populations and a mean seroprevalence is taken including animals from colonies with and without LBV maintenance. Although genetically isolated, if Annobon *E*. *helvum* bats intermittently contact other populations, which would enable introduction of the virus: this could lead to transient circulation of LBV on the island but not long-term population persistence.

Our sensitivity analysis supports the findings from the other RABV systems, that the proportion of bats that become infectious (*ρ*) and the long lyssavirus incubation periods (*σ*) are important for disease maintenance [4]. However, our analyses also suggest that MDA might affect infection maintenance because an increased rate of MDA loss led to a greater likelihood of LBV maintenance in the simulation. Whether MDA is protective or not will be a useful biological question to address in future studies. In our PRCC analysis, disease-induced mortality rate (*α*) also affected LBV maintenance, but less so than the proportion of the population that becomes infectious (*ρ*), the long lyssavirus incubation period (*σ*) or the rate of loss of MDA (ψ).

This quantitative investigation of the natural dynamics of a *Lyssavirus* in African bats not only increases our understanding of these systems, but informs future field and experimental studies. Future studies may test the assumptions in, and results from, these studies. Experimental studies to characterise LBV infection in *E*. *helvum* could help to test assumptions, such as LBV infection always causes mortality in bats [71], that infectious periods are short [72], that seroconversion leads to immunity [55], and that MDA are protective [71]. Given the model was fit to mean, point estimates of seroprevalence with large confidence intervals, the fact that the model captures general features of the system is encouraging, but must be treated with caution. Data with finer temporal resolution and increased sampling of younger age classes in particular will allow the model here to be fitted again and tested more rigorously.

Our model results suggest that in the absence of hibernation, lyssavirus infection prevalence might not be as seasonal as RABV in temperate bat systems [4]. Risk of human infection may be low from individual bats, as our model predicts only 1 in approximately 8,300 bats will be infected at any one time and that the seasonality is dampened relative to what might be expected given the strongly seasonal supply of young bats through birthing. We are mindful that these model predictions require further field data validation; however, our analyses suggest the risk of infection to humans or other species may simply be the presence of *E*. *helvum*, a migratory species, and the human contact rates in each location. Large numbers of *E*. *helvum* are harvested as a source of meat in West Africa, making human-bat contact a common but seasonal occurrence [75–77]. Our model predicts that the seasonal contact in Ghana, for example, occurs when the adult bats have fewest infected individuals. However, the range from the simulations suggests only few adults are infected at any one time throughout the year (Fig 4 and Figures G & H in S1 Supplementary Information). Age specific differences may exist, as our model predicts approximately twice as many juveniles to be infected as adults (Figures G & H in S1 Supplementary Information), which is similar to findings from other systems [78]. Thus zoonotic infection might be further reduced if juvenile-human contact rates are minimised. The results from our work, therefore, are useful for both understanding the mechanisms of maintenance and for understanding the risk to other species. This bat species has been linked to a range of other viruses [79–82], so future studies to compare mechanisms of viral maintenance within this species will be enlightening [44], and may help inform how viruses ‘spill over’ to infect new hosts and when this may occur.

## Supporting information

S1 Supplementary Information Contains: Methods A-B; Figures A-L; Tables A-F; and Supplementary references. (DOCX)

S1 Dataset Contains: Code A-B; Tables A-C & E in Excel Format. (ZIP)
