# Supplementary material for: Maternal antibody and the maintenance of a lyssavirus in populations of seasonally breeding African bats
Source: PLoS One. 2018 Jun 12;13(6):e0198563. doi: 10.1371/journal.pone.0198563 (PMC5997331; doi:10.1371/journal.pone.0198563)
Supplement: S1 Supplementary Information — (DOCX) [file pone.0198563.s001.docx]

S1 Supplementary information – Hayman *et al*

[Method A: Captive study 3](#_Toc515902524)

[Method B: Pathogen extinction - sensitivity analysis 4](#_Toc515902525)

[Figures 6](#_Toc515902526)

[Figure A 6](#_Toc515902527)

[Figure B 7](#_Toc515902528)

[Figure C 8](#_Toc515902529)

[Figure D 9](#_Toc515902530)

[Figure E 10](#_Toc515902531)

[Figure F 11](#_Toc515902532)

[Figure G 12](#_Toc515902533)

[Figure H 13](#_Toc515902534)

[Figure I 14](#_Toc515902535)

[Figure J 15](#_Toc515902536)

[Figure K 16](#_Toc515902537)

[Figure L 17](#_Toc515902538)

[Tables 18](#_Toc515902539)

[Table A 18](#_Toc515902540)

[Table B 19](#_Toc515902541)

[Table C 20](#_Toc515902542)

[Table D 21](#_Toc515902543)

[Table E 22](#_Toc515902544)

[Table F 23](#_Toc515902545)

[Supplementary references 24](#_Toc515902546)

## Method A: Captive study

The details of the enclosure are given elsewhere (Supplementary reference 1), but briefly, an approximately 600m^3^ cage in Accra, was populated with *E. helvum* bats from Accra, each double marked with ball-chain necklaces and with passive integrated transponder (PIT) tags (microchips) subcutaneously (Supplementary reference 1). Initially a pilot study of twelve male bats were kept in captivity for six months, with an additional ten mixed sex and aged bats introduced for a two-month period, before the final mixed group of 53 bats were introduced. The enclosure was then maintained as a closed colony with a starting population of 75 bats and animals allowed to breed freely. Group composition, entry dates and births are shown in Table A. Bats were bled on entry and at recapture. Bat captures were undertaken approximately every 2-4 months. Animals were classified by age (neonate (Neo), juvenile (Juv), sexually immature adult (SI), and sexually mature adult (SM)) and sex (male (M), and female (F)). This age classification is used for the captive bat statistical analysis because despite animals aging in captivity (e.g. SI adults reaching sexual maturity) their serological status was presumed to be defined by their age on entry into the cage (see captive study results and discussion). Serological data available for the 91 individual captive bats are reported in this study are presented in raw and summarised form in this online material.

## Method B: Pathogen extinction - sensitivity analysis

Given the uncertainty of some of the parameter values and specifically to examine the relative importance of different biological aspects of the system, such as the effect of MDA on infection persistence, we performed a sensitivity analysis on model parameters relating to both infection and host demography and how their variation affected LBV persistence. For the sensitivity analysis we used a multi-parameter sensitivity analysis proposed by Blower and Dowlatabadi (Supplementary reference 2). We constructed 100 random parameter sets using stratified random samples from uniform distributions spanning a range of potential values for the parameters of interest (Table 1) using Latin hypercube sampling (lhs package in R, Supplementary reference 3). To determine the sensitivity of changes in birth synchrony, *s,* to the birth rate scalar parameter, *κ*, we varied *s* while fixing the overall birth rate for the year by keeping the integral of the PGF to 0.49. As *κ* was insensitive to changes in *s* (Figure A) we kept *κ* constant for the sensitivity analysis. The range of the parameters was determined by increasing and decreasing the parameter used in our parameter sets by an order of magnitude, with the exception of those for which this would be outside biologically plausible values. Therefore, the number of birth pulses, for example, was restricted to one per year and the number of births limited to a maximum of one per female per year. Each of the 100 parameter sets was simulated 1000 times for 35 years. A 35-year time period was chosen because some parameter combinations for some stochastic simulations took more than 20 years to reach a period of quasi-equilibrium (i.e. regular dynamics on visualisation). Partial-rank correlation coefficients (PRCC) between each parameter and the persistence of infection in the population after 35 year simulations determined the relative importance of each parameter (Supplementary references 2-5). A positive PRCC indicates that as the parameter increases, LBV persistence increases and a negative PRCC indicates LBV persistence decreases with increasing parameter values. Significance of PRCC is determined by a student’s *T* distribution with *N-2* degrees of freedom, where *N* is the number of parameter sets. We checked for non-linear, non-monotonic relationships among the output and the LHS parameter inputs to ensure the assumptions of the PRCC analysis were met (Supplementary reference 5).

In a *K*+1 by *K*+1 symmetric matrix, *C*, where *K* is the number of parameters, and 1 to *N* is the rank of each column defined by the set (*r_1i_, r_2i_, … r_ki_, R_i_*), where *i* = run number, *μ* = the average rank ((1+*N*)/2), the matrix *C* is defined with elements *C_ij_*, such that:

$C_{ij}=\frac{\sum_{t=1}^{N} (r_{it}-\mu)(r_{jt}-\mu)}{\sqrt{\sum_{t=1}^{N} {(r_{it}-\mu)}^{2}\sum_{s=1}^{N} {(r_{js}-\mu)}^{2}}} i,j=1,2, \ldots K.$

*R_i_* replaces *r_ij_* and *r_is_* for the *C_j_*, *_K_*_+1_ elements, and the inverse of C becomes *b_ij_* for matrix *B*. The PRCC between the *i*th parameter and *y*th variable is then:

${PRCC}_{iy}=\frac{{-b}_{i,K+1}}{\sqrt{b_{i}b_{K+1,K+1}}}$

Significance (*t_iy_*) of PRCC*_iy_* is determined by a student’s *T* distribution with *N-2* degrees of freedom, thus:

$t_{iy}={PRCC}_{iy}\sqrt{\frac{N-2}{{1-PRCC}_{iy}}}$

All analyses were performed in R (Supplementary reference 6).

## Figures

Figure A Fitting the periodic Gaussian function to changes in proportion of the pregnant females in an *E. helvum* population (Supplementary reference 7) to estimate the shape parameters (A). Fixing the birth rate for *E. helvum*, and varying *s*, synchrony, from 7 (low synchrony) to 167 (high synchrony) led to very little change in *κ* (1.3 to 1.5), the scalar for the periodic Gaussian function (Supplementary reference 8).

Figure B Changes in anti-Lagos bat virus antibody titers are shown for captive bats used in this study. The ages (Neonate, Juvenile, SI-sexually immature adult, and SM-sexually mature adult) are the ages at which the bats entered the study. All neonates were born in captivity. Raw data are in Table C and here divided by sex.

**
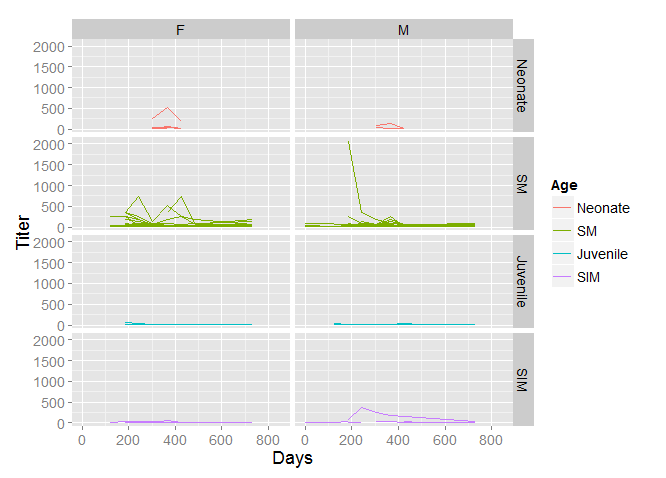
**

Figure C Frequency distributions of log_2_ reciprocal antibody titers for the age-specific serological results from captive bats (see Figure B and Table E). See Figure D for the wild bat titers from Supplementary reference 9 for comparison. 2.4 is negative.

Figure D Frequency distributions of log_2_(reciprocal antibody titers) for the age-specific serological results for wild bats from (Supplementary reference 9) (see Figure C for captive bat data).

Figure E The age-specific seroprevalence of anti-Lagos bat virus (LBV) antibody in 88 *Eidolon helvum* from Ghana (Top, Table E). Data from male and female animals are combined. Numbers of individuals are shown by bubble diameter (size). The age independent force of infection is plotted (Bottom), along with the results from two further models (Table D). An age independent constant force of infection is used in the mechanistic simulation model. These data are a subset of the total wild seroprevalence data the mechanistic model was fit to. 1.6 is negative.


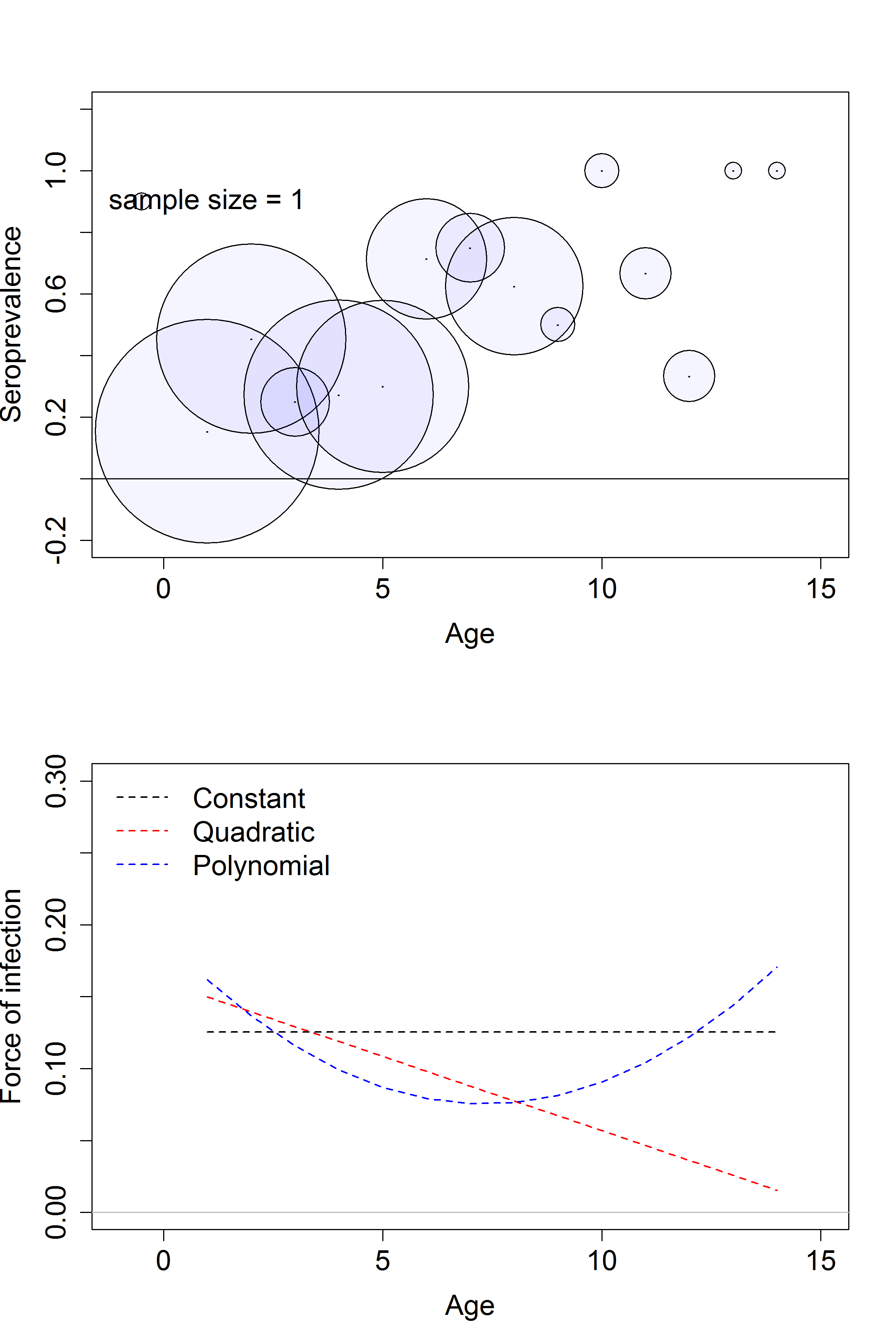


Figure F The likelihood surface for the field seroprevalence data given the stochastic models for a range of *ρ* (the probability of becoming infectious, y axis) and *β* (transmission coefficient, x-axis) values, estimated by particle filtering. Grey lines bisect the surface plot at the best estimates (*β* = 7.6*, ρ* = 0.056, grey lines) and side plots show the two-dimensional plots for individual parameters with the likelihood values shown in cross-section. See Table 1 for the other parameter values.

Figure G Infection dynamics for a single stochastic run from a stochastic *Eidolon helvum* – Lagos bat virus model with maternally-derived antibody, age structure (juvenile and adult), and a seasonal birth pulse. Dynamics for the infected juvenile (y – axis, numbers) and adult age categories are shown. Parameters are in Table 1. The simulation is shown from day 8400 onward to show the dynamics are the initial 20 years initial time steps. Figure H shows the mean values for all 1000 simulations.

Figure H Infection dynamics for a 1000 stochastic runs from a stochastic *Eidolon helvum* – Lagos bat virus (LBV) model with maternally-derived antibody, age structure (juvenile and adult), and a seasonal birth pulse. Dynamics for the adult and juvenile age category are shown for infected classes. Note in approximately 30% of the simulations LBV failed to persist and these are not shown. Mean numbers of infected juveniles (black) and adults (white) are shown. Parameters are in Table 1. Figure G shows a single run from these 1000.

Figure I The observed field seroprevalence data and the mean model anti-Lagos bat virus seroprevalence. Data are shown for juvenile and adult *Eidolon helvum* bats from 1000 simulations of the stochastic model using the *ρ* (the probability of becoming infectious, 0.056) and *β* (transmission coefficient, 7.6) estimated by maximum likelihood. Ninety-five percent confidence intervals are shown for the data and point estimates for the mean from the simulations. See Table 1 for the other parameter values. The correlation between observed and the model results is shown in the inset. The model was allowed to run before fitting to the data and the time in days of the model simulation is given on the x-axis.

Figure J The correlation between the simulated model results and data when the model is fit to data less one time series data point than the 12 available (jackknifing). The observed field seroprevalence data and the mean model anti-Lagos bat virus seroprevalence for juvenile and adult *Eidolon helvum* bats from 1000 simulations of the stochastic model using the *ρ* (the probability of becoming infectious) and *β* (transmission coefficient) estimated by maximum likelihood. See Table 1 for the other parameter values. The correlation between observed and the model results is shown and uses the mean of the 1000 simulations compared to the collected data. Full values are in Table F.


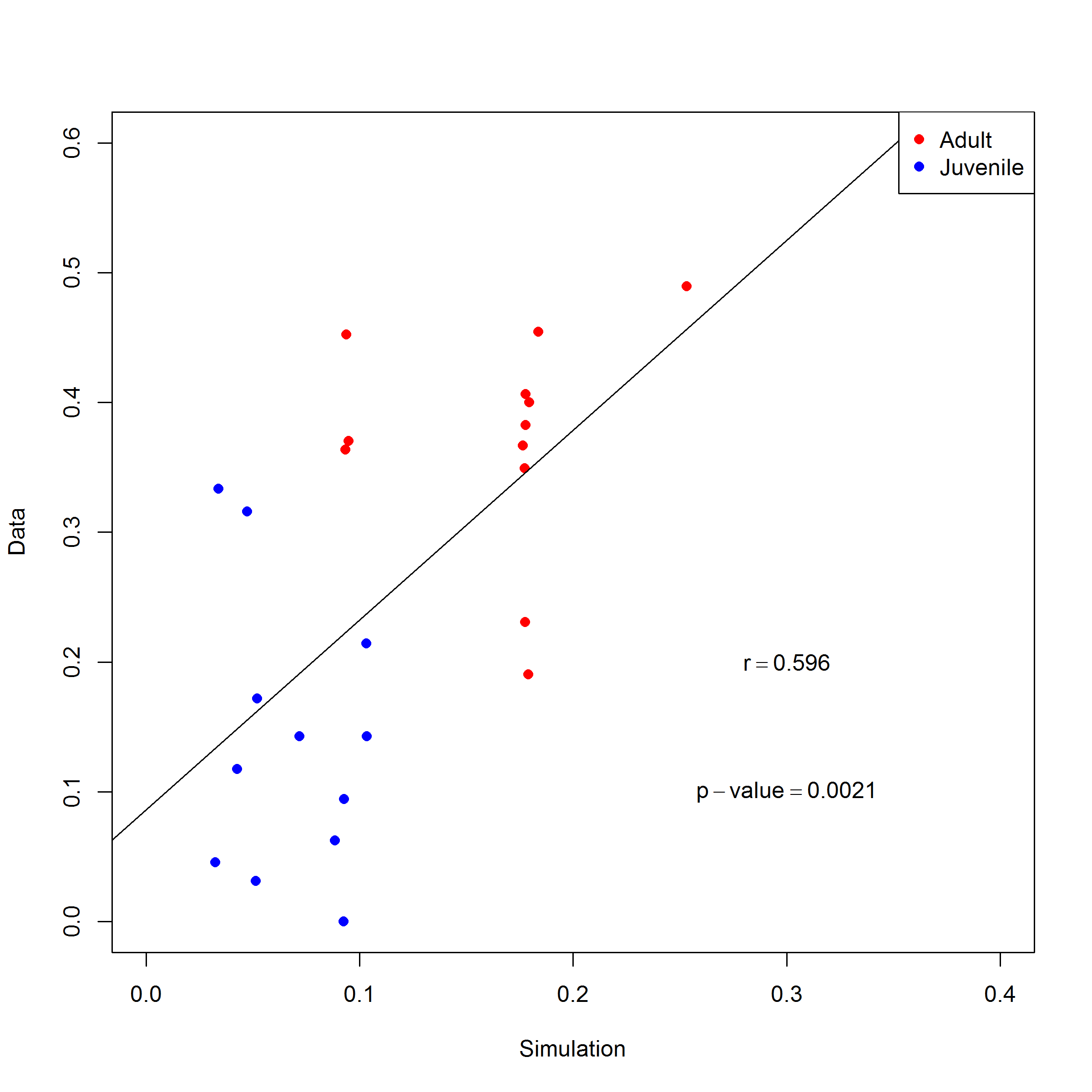


Figure K The standard deviation of the maximum likelihood values for the model fit to the data set using simulated ‘out-of-sample’ cross-validation (Figure J). The model likelihoods and best estimates for the simulated out-of-sample testing are in Table F. This figure shows the uncertainty around the estimates due to the removal of data points and the relative stability of the parameter estimates.


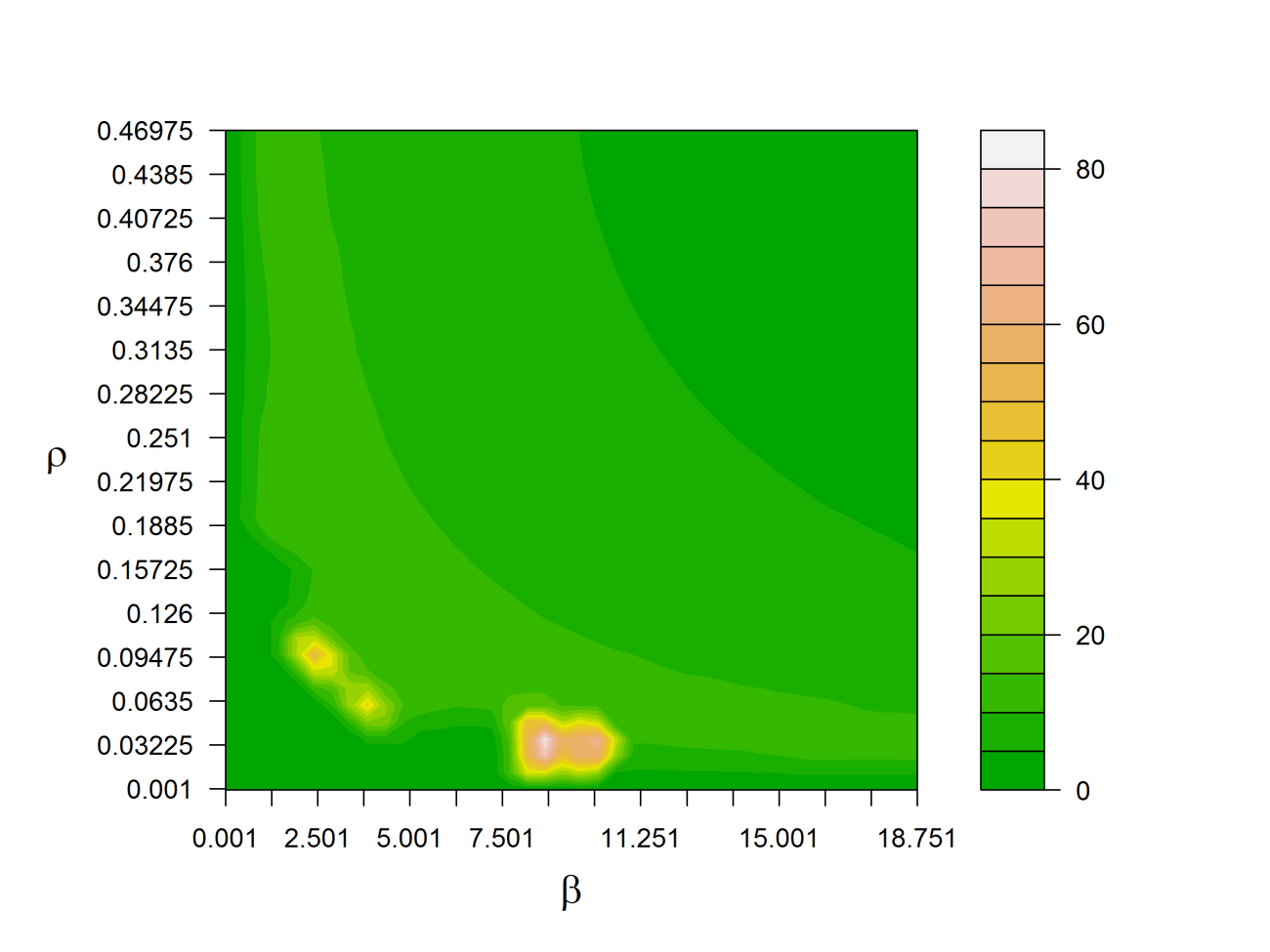


Figure L The distribution of seroprevalence data from 10 countries across sub-Saharan Africa, reported in (Supplementary reference 9).


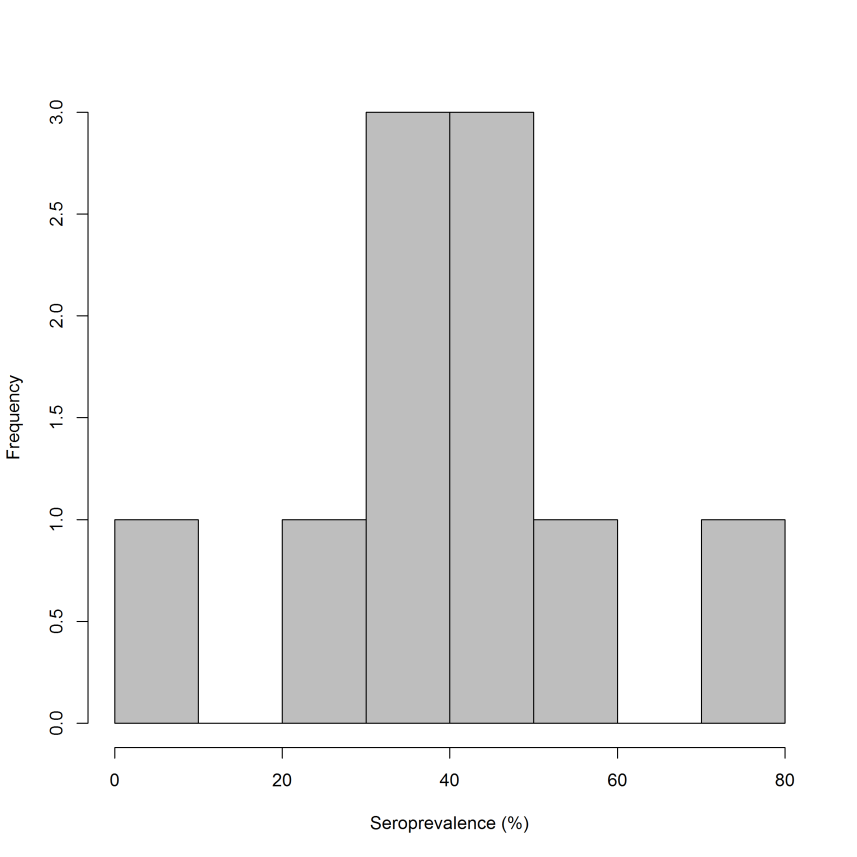


## Tables

Table A Characteristics of *Eidolon helvum* bats in the captive colony of bats in Accra, Ghana. Age is defined by the age at which bats were captured and entered the cage, SI=sexually immature, SM=sexually mature, Juv = juvenile, whereas neonates (Neo) were born in captivity. u is for those juveniles that were not sexed at the time of sampling. Note that there are 115 bats here but serological data were only available for 91 (see main text).

See S1 Dataset file: **Table A**

Table B Longitudinal time series data of the number of bats seropositive against Lagos bat virus in Accra, Ghana (Supplementary reference 10).

See S1 Dataset file: **Table B**

Table C Log_2_ anti-Lagos bat virus antibody titers for captive bats (Table A)

See S1 Dataset file: **Table C**

Table D Akaike information criteria (AIC) values for three models relating to force of infection fit to age specific serological data (Table E, Figure E). AIC, ΔAIC and AIC model weights (w) are given.

| **Model** | **Number of parameters** | **AIC** | **ΔAIC** | **wAIC** |
| --- | --- | --- | --- | --- |
| Age independent model (Supplementary reference 11) | 1 | 37.28 | 0 | 0.58 |
| Quadratic (change point model) (Supplementary reference 12) | 2 | 38.58 | 1.3 | 0.30 |
| Polynomial (Supplementary reference 13) | 3 | 40.39 | 3.11 | 0.12 |

Table E Age, sex, and antibody titers for wild *Eidolon helvum*, using ages specific data (Supplementary references 9 & 10) (see Figure E).

See S1 Dataset file: **Table E**

Table F Summaries of the model likelihoods and best estimates for the simulated out-of-sample testing as described in the main text. SP is seroprevalence, A adult, J juvenile, Sim is the model simulation prediction and Data the observed data. Beta, Rho and MLL are *β* (transmission coefficient), *ρ* (the probability of becoming infectious) and the MLE.

| **Data point removed** | **Sim.SPA** | **Sim.SPJ** | **Data.SPA** | **Data.SPJ** | **Beta** | **Rho** | **MLL** |
| --- | --- | --- | --- | --- | --- | --- | --- |
| **1** | 0.094956 | 0.033985 | 0.37037 | 0.333333 | 12.501 | 0.03225 | -265.988 |
| **2** | 0.093329 | 0.032521 | 0.363636 | 0.045455 | 12.501 | 0.03225 | -248.475 |
| **3** | 0.093866 | 0.042633 | 0.452381 | 0.117647 | 12.501 | 0.03225 | -255.469 |
| **4** | 0.179544 | 0.103173 | 0.4 | 0.214286 | 13.751 | 0.03225 | -263.794 |
| **5** | 0.177741 | 0.051518 | 0.382716 | 0.03125 | 13.751 | 0.03225 | -233.789 |
| **6** | 0.176515 | 0.071818 | 0.366667 | 0.142857 | 13.751 | 0.03225 | -259.068 |
| **7** | 0.177666 | 0.092723 | 0.406504 | 0.09434 | 13.751 | 0.03225 | -234.691 |
| **8** | 0.178968 | 0.103452 | 0.190476 | 0.142857 | 13.751 | 0.03225 | -253.061 |
| **9** | 0.183605 | 0.047378 | 0.454545 | 0.315789 | 13.751 | 0.03225 | -251.899 |
| **10** | 0.177463 | 0.052074 | 0.230769 | 0.171875 | 13.751 | 0.03225 | -241.729 |
| **11** | 0.253204 | 0.088582 | 0.489583 | 0.0625 | 6.251 | 0.0635 | -243.42 |
| **12** | 0.177273 | 0.092612 | 0.349398 | 0 | 13.751 | 0.03225 | -231.203 |

## Supplementary references

1. Baker KS, Suu-Ire R, Barr J, Hayman DT, Broder CC, Horton DL, et al. Viral antibody dynamics in a chiropteran host. The Journal of animal ecology. 2014 Mar;83(2):415-28. PubMed PMID: 24111634. Pubmed Central PMCID: 4413793.

2. Blower SM, Dowlatabadi H. Sensitivity and uncertainty analysis of complex-models of disease transmission - an HIV model, as an example. International Statistical Review. 1994 Aug;62(2):229-43. PubMed PMID: ISI:A1994PB04800004. English.

3. Carnell R. lhs: Latin Hypercube Samples. 0.5 ed2009.

4. Buhnerkempe MG, Eisen RJ, Goodell B, Gage KL, Antolin MF, Webb CT. Transmission shifts underlie variability in population responses to Yersinia pestis infection. PLoS One. 2011;6(7):e22498. PubMed PMID: 21799873. Pubmed Central PMCID: 3143141.

5. Marino S, Hogue IB, Ray CJ, Kirschner DE. A methodology for performing global uncertainty and sensitivity analysis in systems biology. Journal of Theoretical Biology. 2008 Sep 7;254(1):178-96. PubMed PMID: 18572196. Pubmed Central PMCID: 2570191.

6. R Development Core Team. R: A language and environment for statistical computing. 2012. Epub 2.15.1.

7. Mutere FA. The breeding biology of equatorial vertebrates; reproduction in the fruit bat *E. helvum*, at latitude 0°20'N. Journal of Zoology. 1967;153:153-61.

8. Hayman DT. Biannual birth pulses allow filoviruses to persist in bat populations. Proc. R. Soc. B. 2015 Mar 22;282(1803):20142591. PubMed PMID: 25673678. Pubmed Central PMCID: 4345444.

9. Peel AJ, Baker KS, Hayman DTS, Suu-Ire R, Breed AC, Gembu G-C, Lembo T, Fernández-Loras A, Sargan DR, Fooks AR, et al. Bat trait, genetic and pathogen data from large-scale investigations of African fruit bats, *Eidolon helvum*. Scientific data. 2016 3: 160049

10. Hayman DT, Fooks AR, Rowcliffe JM, McCrea R, Restif O, Baker KS, et al. Endemic Lagos bat virus infection in Eidolon helvum. Epidemiology and infection. 2012 Dec;140(12):2163-71. PubMed PMID: 22370126.

11. Muench H. Derivation of Rates from Summation Data by the Catalytic Curve. J Am Stat Assoc. 1934 Mar;29(185):25. PubMed PMID: WOS:000188341800003. English.

12. Griffiths DA. Catalytic Model of Infection for Measles. J Roy Stat Soc C-App. 1974;23(3):330-9. PubMed PMID: WOS:A1974V708700008. English.

13. Grenfell BT, Anderson RM. The estimation of age-related rates of infection from case notifications and serological data. The Journal of Hygiene. 1985 Oct;95(2):419-36. PubMed PMID: 4067297. Pubmed Central PMCID: 2129533. English.
